# Preferential Orientation of Photochromic Gadolinium Oxyhydride Films

**DOI:** 10.3390/molecules25143181

**Published:** 2020-07-12

**Authors:** Elbruz Murat Baba, Jose Montero, Dmitrii Moldarev, Marcos Vinicius Moro, Max Wolff, Daniel Primetzhofer, Sabrina Sartori, Esra Zayim, Smagul Karazhanov

**Affiliations:** 1 Department for Solar Energy, Institute for Energy Technology, NO-2027 Kjeller, Norway; dmitry.moldarev@physics.uu.se (D.M.); Smagul.Karazhanov@ife.no (S.K.); 2 Nanoscience & Nano Engineering Department, Istanbul Technical University, 34469 Istanbul, Turkey; ozesra@itu.edu.tr; 3 Department of Materials Science and Engineering, The Ångström Laboratory, Uppsala University, SE-75121 Uppsala, Sweden; jose.montero-amenedo@angstrom.uu.se; 4 Department of Physics and Astronomy, Uppsala University, Box 516, 751 20 Uppsala, Sweden; marcos.moro@physics.uu.se (M.V.M.); max.wolff@physics.uu.se (M.W.); daniel.primetzhofer@physics.uu.se (D.P.); 5 Department of Materials Science, National Research Nuclear University (MEPhI), Kashirskoe shosse 31, 115409 Moscow, Russia; 6 Department of Technology Systems, University of Oslo, NO-2027 Kjeller, Norway; sabrina.sartori@its.uio.no; 7 Physics Engineering Department, Istanbul Technical University Faculty of Science and Letters, 34469 Istanbul, Turkey

**Keywords:** gadolinium oxyhydride, rare earth metal oxyhydride, mixed anion materials, photochromic effect, preferential orientation, band gap

## Abstract

We report preferential orientation control in photochromic gadolinium oxyhydride (GdHO) thin films deposited by a two-step process. Gadolinium hydride (GdH_2-x_) films were grown by reactive magnetron sputtering, followed by oxidation in air. The preferential orientation, grain size, anion concentrations and photochromic response of the films were strongly dependent on the deposition pressure. The GdHO films showed a preferential orientation along the [100] direction and exhibited photochromism when synthesized at deposition pressures of up to 5.8 Pa. The photochromic contrast was larger than 20% when the films were deposited below 2.8 Pa with a 0.22 H_2_/Ar flow ratio. We argue that the relation of preferential orientation and the post deposition oxidation since oxygen concentration is known to be a key parameter for photochromism in rare-earth oxyhydride thin films. The experimental observations described above were explained by the decrease of the grain size as a result of the increase of the deposition pressure of the sputtering gas, followed by a higher oxygen incorporation.

## 1. Introduction

Rare-earth metal oxyhydride (REMOH) thin films have attracted increasing interest during recent years due to their reversible photochromic properties at room temperature and ambient pressures [1,2,3,4]. Until now, the structure, chemistry and many properties of oxyhydrides have yet to be fully explored and almost no data is available in the usual material databases for rare-earth oxyhydrides [5]. Having multi-anion structures, rare-earth oxyhydrides present a high level of flexibility for material development thanks to the combination possibilities provided by two different anions: hydride and oxide. Besides powder production with a tube furnace [6,7], rare-earth metal oxyhydride films have also been successfully produced by reactive magnetron sputtering [2,8,9] and e-beam evaporation [10]. Typically, the synthesis consists of few steps. First, a di-hydride precursor film [3,8,9] is deposited onto a substrate (e.g., glass). It is then oxidized by exposure to air, resulting in photochromic polycrystalline oxyhydride films [11]. Several studies further attempted at each time to reveal the mechanisms of photochromism in rare-earth metal oxyhydrides through time resolved X-ray diffraction using synchrotron radiation [12], composition analysis [3,9,13] and positron annihilation spectroscopy (PAS) [14,15]. However, the answer for the exact mechanism for rare-earth metal oxyhydrides still debated.

The first study of gadolinium with varying levels of oxygen and hydrogen (GdO_y_H_x_) was carried out by Miniotas et al. [16], who reported gigantic resistivity, as well as band gap differences arising from compositional variations, i.e., a different hydrogen to oxygen ratio. However, the discovery of the photochromic properties of gadolinium oxyhydride (GdHO) had to wait until recently, when Nafezarefi et al. [2] reported GdHO films exhibiting photochromic contrast (differences in optical transmittance in dark and bleached state) around 45% after 8 h of illumination.

Generally, photochromic YHO thin films show a preferred growth along the [100] direction [12,17]. Phase diagrams and structural analysis have been reported for different REMOH (REM = Y, Sc or Gd) compounds, YHO being the most studied system. These studies claim that the fcc structure and phase diagram for YHO can be extrapolated for other rare-earth metal oxyhydrides [9]. However, the correlation between crystalline orientation and photochromic performance has not been investigated. Different crystal orientations might facilitate or hinder transport phenomena, as it did in a previous study [18] where oxygen transport into the material was demonstrated to play an important role for the evolution of photochromic properties of YHO.

In the present paper, we studied how the optical, compositional and structural properties of photochromic GdHO thin films are affected by subtle changes in preferential growth, which are controllable as a function of the deposition parameters.

## 2. Results

**Figure 1:**
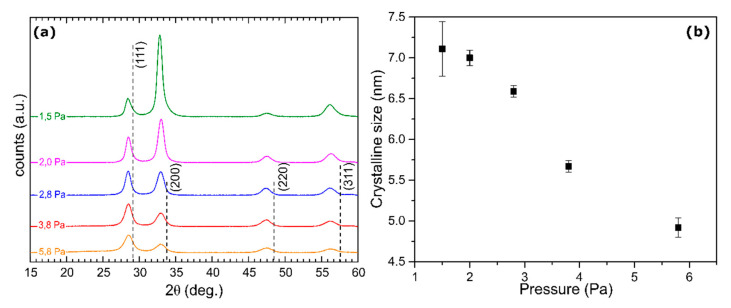
(**a**) Grazing-incidence X-ray diffraction (GIXRD) results for samples deposited at 1.5, 2.0, 2.8, 3.8 and 5.8 Pa. Photochromic films exhibited a change in the relative intensity of the different diffraction peaks as the oxygen incorporation increased due to the increase in deposition pressure. (**b**) The grain size reduced as a result of increased deposition pressure.

Figure 1a shows the grazing-incidence X-ray diffraction (GIXRD) patterns for photochromic GdHO deposited at different *p* values measured under ambient air. The intensity parameter δ achieved its lowest and highest value for samples deposited at 5.8 Pa (δ = 0.328) and 1.5 Pa (δ = 0.818), respectively. All films showed preferential growth along the [100] direction when compared to the standard diffraction peaks of GdH_2_ (Joint Committee of Powder Diffraction Standards JCPDS, card number 00-050-1107). This reference standard pattern, where δ = 0.2537, is depicted as vertical lines in Figure 1a. The average grain sizes deduced using the diffraction peaks (111), (200), (220) and (311) are plotted in Figure 1b as function of P. The grain size decreased with the increasing pressure P: the dependence was weak at P and below 2 Pa, was much stronger at intermediate pressures, 2 < *p* < 5 Pa and diminished as P exceeded 5 Pa. It is well established through the Thornton diagram [19,20,21,22] that sputter deposition at higher pressures results in the opening of inter-grain boundaries. Open inter-grain boundaries in the precursor gadolinium hydride films resulted in a higher surface area and faster oxidation kinetics. Consequently, the precursor hydride films deposited at higher pressures resulted in oxyhydrides with a higher oxygen content. Time-of-flight energy elastic recoil detection analysis (ToF-E ERDA) (Figure S3) confirmed a higher oxygen content in samples deposited at higher *p*, which resulted in films with a wider band gap (Table S1). The band gap values of all samples are presented in Table S1. With increased deposition pressure, the band gap increased by 1 eV, approaching the value for Gd_2_O_3_. The presence of a Gd_2_O_3_ phase can explain the band gap widening (Table S1) and the reduced photochromic response (Figure S2) observed in samples obtained from precursor hydrides deposited at higher *p*.

Previous studies performed on YHO [23] suggested that variations of the band gap as a function of *p* were the result of changes in the O content in the films. Later, the oxygen incorporation result with the increased band gap by controlling deposition pressure was established [24] through compositional analysis performed on YHO samples deposited at 1 Pa and 6 Pa. It was shown that oxygen and hydrogen are anticorrelated.

**Figure 2:**
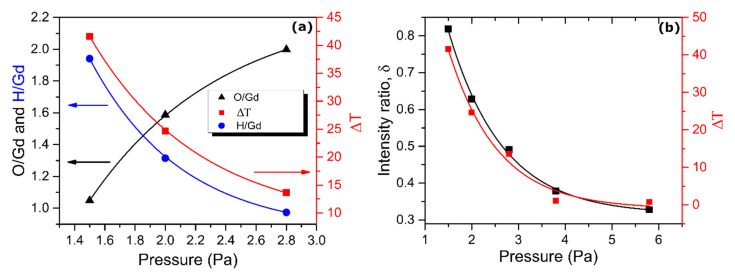
(**a**) Composition of photochromic GdHO films and optical contrast ΔT as a function of deposition pressures. Optical contrast ΔT is averaged over wavelength 550 nm to 1000 nm. (**b**) Intensity ratio δ and ΔT as a function of deposition pressure. Curves are visual guides.

The compositional analysis performed in the present work also proved the anti-correlation between the O and H contents in the films. Figure 2a presents the O and H ratio as a function of *p*. Samples deposited at larger pressures were not included, as they presented low or no photochromism (Figure S2). The full depth profiles of oxygen and hydrogen can be found in the supplementary material, Figure S3. Figure 2a also displays the dependence of the photochromic contrast ΔT on *p*. Analysis shows strong correlation between *p*, H content and ΔT, which is consistent with earlier findings [3] for YHO.

Figure 2b displays the dependence of δ and ΔT on *p*. By increasing *p* from 1.5 to 5.8 Pa, ΔT dropped from 40% to 0%, following the reduction of the intensity parameter δ. This decay with respect to deposition pressure clearly showed the strong correlation between preferential growth, optical properties, chemical composition and grain size of the GdHO films. The films with (200) orientation exhibit higher photochromic contrasts than the films with (111) orientation. It is well known for other oxides [25,26] as well as oxyhydrides [3] that changes in stoichiometry, specifically in oxygen content, result in changes in crystalline orientation and band gap. The experimental observations described above can be explained by a regulation of oxygen uptake through preferential orientation along the [100] direction.

It should be noted that the changes in the preferential growth can also be observed in yttrium oxyhydride (YHO) as a function of film thickness in films deposited under the same *p* [27]. However, preferential orientation was only reported at larger thicknesses (>~200 nm) with no indication of preferential growth in thinner films. According to our study, the lack of preferential growth along the [100] direction should result in smaller grain sizes and a low photochromic response when the film thickness is below 200 nm, in agreement with previous observations [27].

## 3. Materials and Methods

GdHO thin films with thicknesses ranging between 525 and 615 nm were reactively sputtered onto soda-lime glass substrates following a two-step deposition process [3,8,9]. First, gadolinium hydride (GdH_2-x_) thin films were deposited by reactive pulsed DC magnetron sputtering from a metallic Gd target (purity 99.9%) in an H_2_/Ar atmosphere using a Leybold Optics A550V7 sputtering unit (Alzenau, Germany). The discharge power density was 1.33 W/cm^2^ and the hydrogen-to-argon flow ratio (H_2_/Ar) was kept at 0.22. The deposition pressure *p* was varied from 1.5 to 5.8 Pa by adjusting a throttle valve placed between the deposition chamber and the vacuum line. The deposition process was carried out without an intentional heating of the substrate. In a second step, the GdH_2-x_ films were oxidized when they were removed from the chamber and were exposed to air, thus turning into a transparent and photochromic GdHO.

The thickness of the films was measured using a Step-200 profilometer (Milpitas, CA, USA). The crystallographic structures of the films were characterized by grazing-incidence x-ray diffraction (GIXRD) in a Bruker Siemens D5000 (CuKα radiation, parallel beam geometry and 2° angle of incidence, (Billerica, MA, USA). Grain sizes were calculated using the Scherrer equation [28], assuming spherical crystals;

(1) L=Kλ/βcosθ,

where *L* is the grain size, *K* is the shape factor chosen as 0.9, *λ* is the wavelength, *β* is the peak broadening at half maximum and *θ* is the diffraction angle.

Further, we defined the intensity ratio parameter δ [25] as:

(2) $$\delta =I_{\left(200\right)}/\left[I_{\left(111\right)}+I_{\left(200\right)}\right],$$

where *I*_(111)_ and *I*_(200)_ are the intensities of the [111] and [100] diffraction maxima.

Optical transmittance T, reflectance R and absorptance A of the GdHO films in the clear and photo-darkened state were obtained with an integrated sphere using an Ocean Optics QE6500 spectrophotometer (Dunedin, FL, USA). The photochromic contrast was defined as ΔT = T_clear_ − T_dark_, where T_clear_ and T_dark_ were the transmittance in the clear and photo-darkened state, respectively. Analogously, the photochromic contrast in A and R were defined as ΔA = A_clear_ − A_dark_ and ΔR = R_clear_ − R_dark_.

The photochromic effect was triggered by a 6 W-lamp (wavelength 405 nm). Cyclic illumination was performed using a 405 nm-laser (4.5 mW). The absorption coefficient *α* was calculated from T and R values;

(3) $$\alpha =d^{-1}\ln\left(\left(1-R^{2}\right)/T\right),$$

where *d* is the film thickness. The absorption coefficient is related to the energy *E_g_* of the optical band gap [29]:

(4) $$\alpha h\nu^{m}=\alpha_{0}\left(h\nu -E_{g}\right),$$

where *hv* is the energy of incident photon, $\alpha_{0}$ is a constant and *m* is a factor that depends on the optical transition (*m* = 2 for direct-allowed and 1/2 for indirect-allowed transitions) [30].

Chemical composition analysis was performed by coincidence time-of-flight energy elastic recoil detection analysis (ToF-E ERDA) at the Tandem Laboratory of Uppsala University. In this work, projectiles of 36 MeV 127I8+ were used as a probe beam and the recoiled specimens were detected by a telescope ToF-E tube placed at 45° with respect to the beam’s direction [31]. A detailed discussion about the ToF-E ERDA setup used on similar samples (YHO) can be found elsewhere [13].

## 4. Conclusions

We studied GdHO films deposited onto glass substrates in an H_2_/Ar plasma prepared at different sputtering pressures. The influence of deposition parameters and film composition on the photochromic performance was systematically studied. We demonstrated that the preferential growth of GdHO films can be controlled by the variation of the deposition pressure. Highly oriented (200) films were formed at deposition pressures ≥1.5 Pa. The photochromic contrast >20% was found for films deposited at ≤2.8 Pa. Within this study, we also established band gap control from 2.8 eV to 3.7 eV over reactively sputtered gadolinium oxyhydride films. We revealed an inverse correlation between deposition pressure and photochromic contrast in GdHO, determined by the oxygen content. Higher deposition pressures during the fabrication of the hydrides resulted in a higher degree of oxygen incorporation, followed by the reduced photochromic response when the sample was exposed to air, forming the oxyhydride. We also showed that increasing oxygen incorporation is related to the preferential orientation changes of the lattice from [100] towards the [111] direction.

## Supplementary Materials

The followings are available online. Figures S1: Transmittance (**a**) and absorptance (**b**) of photochromic gadolinium oxyhydride films 25 reactively sputtered at deposition pressures between 1.5 and 5.8 Pa. Figure S2: Change in (**a**) transmittance, (**b**) reflectance and (**c**) absorptance of samples plotted versus 43 wavelength, deposited between 1.5 Pa and 5.8 Pa before and after 60 minutes of illumination. (**d**) 44 Absorptance and photochromic response, averaged between 550–1000 nm, of samples plotted versus 45 pressure. Figure S3: Depth profiles of (**a**) hydrogen and (**b**) oxygen plotted versus deposition pressure 58 deduced from Tof-E ERDA coincidence spectra (not whown). Table S1: Bandgap values of samples deposited between 1.5 Pa and 5.8 pa; where E_g_^dir^ refers to the 32 energy of the direct bandgap.
