# Supplementary material for: Preferential Orientation of Photochromic Gadolinium Oxyhydride Films
Source: Molecules. 2020 Jul 12;25(14):3181. doi: 10.3390/molecules25143181 (PMC7397192; doi:10.3390/molecules25143181)
Supplement: Supplementary file 1 [file molecules-25-03181-s001.pdf]

molecules

MDPI

Supporting Information

Preferential Orientation of Photochromic Gadolinium Oxyhydride Films

Elbruz Murat Baba[1,2*], Jose Montero[3], Dmitrii Moldarev[1,4,6], Marcos Vinicius Moro [4], Max Wolff[4,6], Daniel Primetzhofer[4], Sabrina Sartori[5], Esra Zayim[2,7] and Smagul Karazhanov[1,6]

[1] Department for Solar Energy, Institute for Energy Technology, NO-2027 Kjeller, Norway;
[2] Nanoscience & Nano Engineering Department, Istanbul Technical University, 34469 Istanbul, Turkey
[3] Department of Materials Science and Engineering, The Ångström Laboratory, Uppsala University, SE-75121 Uppsala, Sweden
[4] Department of Physics and Astronomy, Uppsala University, Box 516, 751 20 Uppsala, Sweden
[5] Department of Technology Systems, University of Oslo, NO-2027, Kjeller, Norway
[6] Department of Materials Science, National Research Nuclear University (MEPhI), 115409 Kashirskoe shosse 31, Moscow, Russia
[7] Istanbul Technical University Faculty of Science and Letters, Physics Engineering Department, Istanbul 34469, Turkey
* Correspondence: Elbruz.Baba@ife.no

Received: date; Accepted: date; Published: date

Optical Characterization

Figure S1 shows (a) optical transmittance T and (b) absorptance A as a function of wavelength λ and photon energy E, respectively, for photochromic GdHO samples (clear state) deposited at pressures P ranging from 1.5 to 5.8 Pa. As P increases, T increases in the visible regime between 400 and 700 nm (a) and the absorption edge, located at shorter wavelengths, shifts towards higher energies (b).

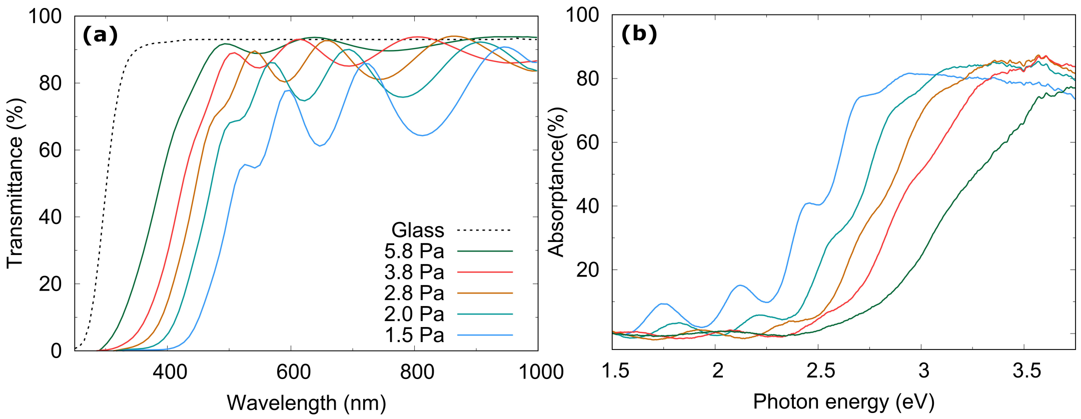

Figure S1. Transmittance (a) and absorptance (b) of photochromic gadolinium oxyhydride films reactively sputtered at deposition pressures between 1.5 and 5.8 Pa.

Figure S2 shows the difference in (a) transmittance ΔT, (b) absorptance ΔA and (c) reflectance ΔR for films deposited at different P before and after illumination. The absorption edge shifts towards shorter wavelengths with increasing P indicating a widening of the bandgap (bandgap energies are tabulated in Table S1). E_g^{dir} increases from 2.8 to 3.7 eV as P increases from 1.5 to 5.8 Pa.

Table S1. Bandgap values of samples deposited between 1.5 Pa and 5.8 pa.; where E_g^{dir} refers to the energy of the direct bandgap.

Sample	Deposition pressure (Pa)	Film Thickness (nm)	E_g^{dir} (eV)
GdH_3			2.4[1]
GdHO	1.5	616	2.8
	2.0	575	3.0
	2.8	550	3.1
	3.8	525	3.3
	5.8	540	3.7
Gd_2O_3			5.4[2]

Figure S2 (d) shows the absorptance averaged between 550 nm and 1000 nm in the clear A_{clear} and photodarkenened A_{dark} states, as well as the photochromic response $|\Delta A|$ as a function of deposition pressure. The photochromic response decreases as P increases (e.g., P = 1.5 Pa, $|\Delta A|$ = 47 % and P = 5.8 Pa, $|\Delta A| \approx 0$ %). These results, which are consistent with previous reports [3], are attributed to the increase of oxygen content in the films prepared at larger P, resulting in higher porosity [4], favoring thus the exchange of hydrogen and oxygen once the samples are removed from the sputtering chamber and exposed to ambient conditions [5].

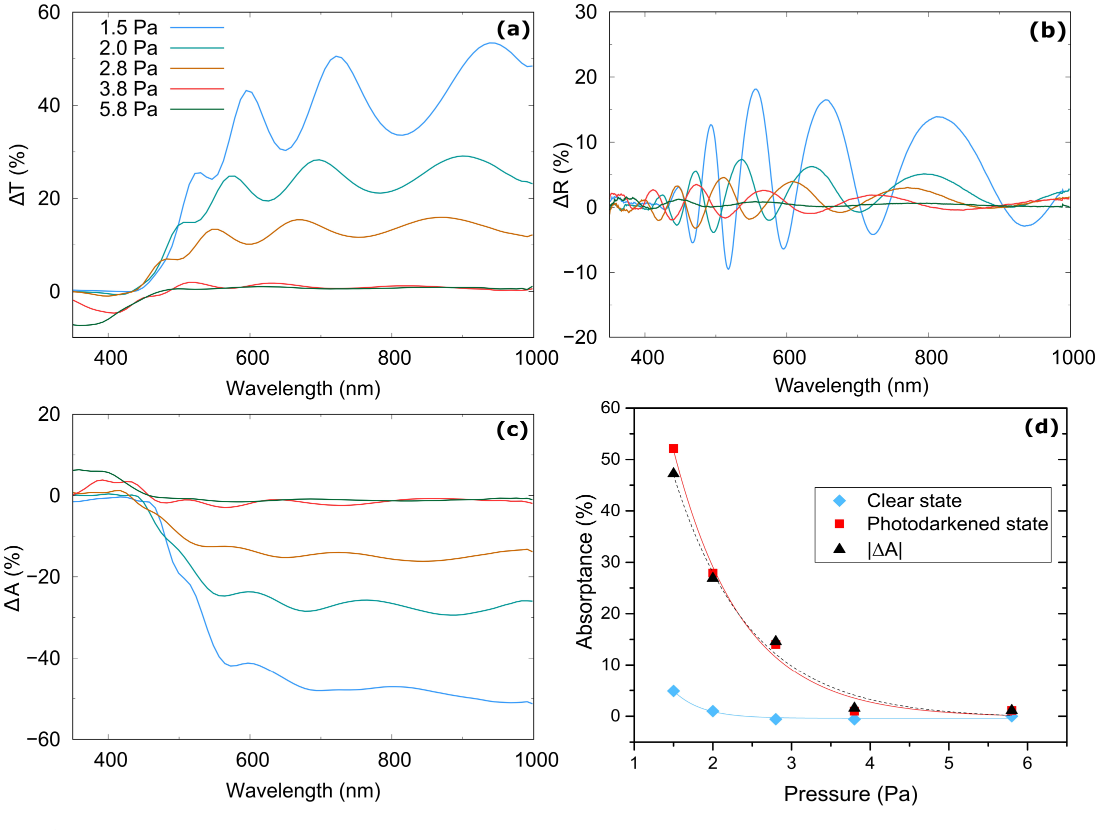

Figure S2. Change in (a) transmittance, (b) reflectance and (c) absorptance of samples plotted versus wavelength, deposited between 1.5 Pa and 5.8 Pa before and after 60 minutes of illumination. (d) Absorptance and photochromic response, averaged between 550-1000 nm, of samples plotted versus pressure.

Composition analysis

Figure S3 (a and b) shows normalized and interpolated maps of the O and H content as a function of deposition pressure and depth. C impurities of up to 4 at. % are not indicated. All the majority elements (i.e., Gd, O and H) are uniformly distributed throughout the films, except at the surface, where an oxygen-rich layer (within 5-10 nm) is found. The increase in deposition pressure

leads to stronger oxidation and decrease of hydrogen content. This effect can be attributed to variations of the porosity of the films [4]. The replacement of H atoms by O atoms during the oxidation process is suggested by the anti-correlation in the O and H content (Figure S3). Typical uncertainties - statistical and systematic - involved in this measurement, especially towards lighter elements as H, are discussed in detail using similar system elsewhere [6].

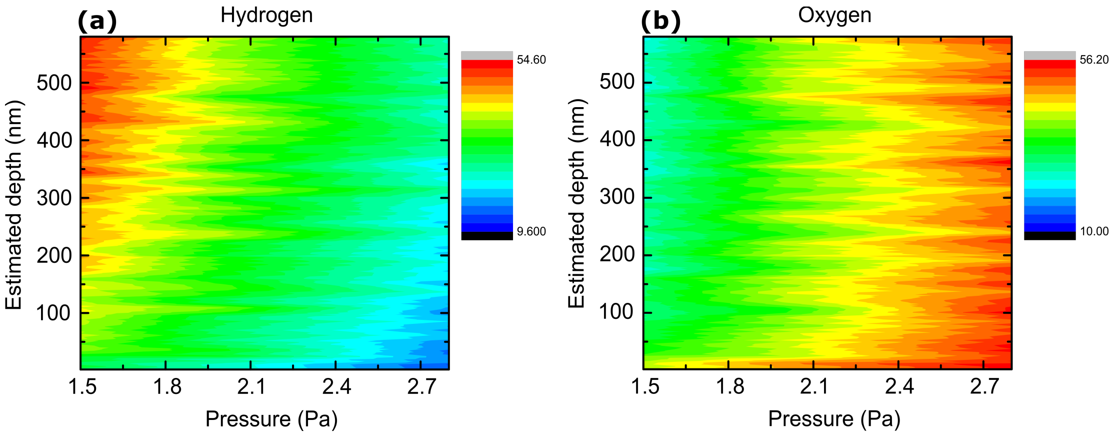

Figure S3. Depth profiles of (a) hydrogen and (b) oxygen plotted versus deposition pressure deduced from Tof-E ERDA coincidence spectra (not whown).

References

1. Miniotas, A.; Hjorvarsson, B.; Douysset, L.; Nostell, P. Gigantic resistivity and band gap changes in GdOyHx thin films. *Appl. Phys. Lett.* **2000**, *76*, 2056–2058, doi:10.1063/1.126253.
2. Adachi, G.Y.; Imanaka, N. The binary rare earth oxides. *Chem. Rev.* **1998**, *98*, 1479–1514, doi:10.1021/cr940055h.
3. You, C.C.; Mongstad, T.; Maehlen, J.P.; Karazhanov, S. Engineering of the band gap and optical properties of thin films of yttrium hydride. *Appl. Phys. Lett.* **2014**, *105*, 1–5, doi:10.1063/1.4891175.
4. Thornton, J.A. High Rate Thick Film Growth. *Annu. Rev. Mater. Sci.* **1977**, *7*, 239–260, doi:10.1146/annurev.ms.07.080177.001323.
5. Moldarev, D.; Primetzhofer, D.; You, C.C.; Karazhanov, S.Z.; Montero, J.; Martinsen, F.; Mongstad, T.; Marstein, E.S.; Wolff, M. Composition of photochromic oxygen-containing yttrium hydride films. *Sol. Energy Mater. Sol. Cells* **2018**, *177*, 66–69, doi:10.1016/j.solmat.2017.05.052.
6. Moro, M.V.; Moldarev, D.; You, C.C.; Baba, E.M.; Karazhanov, S.Z.; Wolff, M.; Primetzhofer, D. In-situ composition analysis of photochromic yttrium oxy-hydride thin films under light illumination. *Sol. Energy Mater. Sol. Cells* **2019**, *201*, 110119, doi:10.1016/j.solmat.2019.110119.
